# Wearable piezoelectric mass sensor based on pH sensitive hydrogels for sweat pH monitoring

**DOI:** 10.1038/s41598-020-67706-y

**Published:** 2020-07-02

**Authors:** E. Scarpa, V. M. Mastronardi, F. Guido, L. Algieri, A. Qualtieri, R. Fiammengo, F. Rizzi, M. De Vittorio

**Affiliations:** 1 Center for Biomolecular Nanotechnologies, Istituto Italiano di Tecnologia, Via Barsanti 14, 73010 Arnesano, Italy; 2 Dipartimento di Ingegneria dell’Innovazione, Università del Salento, Via per Monteroni snc, 73100 Lecce, Italy

**Keywords:** Biosensors, Health care

## Abstract

Colorimetric and electrochemical (bio)sensors are commonly employed in wearable platforms for sweat monitoring; nevertheless, they suffer from low stability of the sensitive element. In contrast, mass-(bio)sensors are commonly used for analyte detection at laboratory level only, due to their rigidity. To overcome these limitations, a flexible mass-(bio)sensor for sweat pH sensing is proposed. The device exploits the flexibility of piezoelectric AlN membranes fabricated on a polyimide substrate combined to the sensitive properties of a pH responsive hydrogel based on PEG-DA/CEA molecules. A resonant frequency shift is recorded due to the hydrogel swelling/shrinking at several pH. Our device shows a responsivity of about 12 kHz/pH unit when measured in artificial sweat formulation in the pH range 3–8. To the best of our knowledge, this is the first time that hydrogel mass variations are sensed by a flexible resonator, fostering the development of a new class of compliant and wearable devices.

## Introduction

In recent years, advances in the fabrications methods of micro and nano electromechanical systems (MEMSs and NEMSs) and the availability of new (bio)sensing platforms have allowed the commercialization of wearable and portable (bio)sensors for checking health status^1–4^. Indeed, such microsystems can continuously monitor the physiological conditions by tracking physical (e.g. heart rate, blood pressure and temperature) and/or chemical parameters (biologically relevant molecules) in a non-invasive way^5,6^. These devices show the advantage to instantly detect the analytes in naturally secreted body fluids, overcoming some limitations of current diagnostic and monitoring methods, such as sampling and storage of samples. Among biofluids, sweat is one of best candidates for continuous and non-invasive wearable (bio)sensing^7^. Sweat is secreted locally (and on-demand) and is directly collected on several sampling areas of the skin, preventing the events of analyte contamination and degradation, which may happen during traditional sample collection and/or storing^8–10^. Sweat contains a wide range of analytes such as metabolites (lactate, glucose, urea, amino acids, etc.), electrolytes (sodium, chloride, potassium, etc.), xenobiotics, antigens, antibodies, ethanol and drugs, whose composition changes can be correlated with pathological conditions or diseases^10^. For example, cystic fibrosis is identified by detecting high chloride levels in sweat^11^. One of the most common parameters to describe the individual health status is sweat pH, whose variations happen both in physiological and pathological conditions. Physiologically sweat pH ranges from 4.0 to 6.8 for healthy subjects^12,13^: for example an increase of sweat pH usually happens during physical activities or in dehydration conditions, when ammonium concentration increases in the fluid. In the case of pathological conditions such as for patients with cystic fibrosis, sweat show a pH value up to 9, due to lack of reabsorption of bicarbonate^14^. Therefore, the changes of sweat pH can be correlated to several physiological and pathological conditions, resulting as one of the most important parameters to be tracked by wearable devices^15,16^. Several chemical (bio)sensors for sweat were developed exploiting electrochemical and colorimetric detection methods. Although these methods are commonly employed to fabricate very selective and sensitive (bio)sensors, they show some drawbacks linked to the sensor’s reusability^17^. In particular, the stability over time of the responsive element, commonly a biological molecule, is affected by environmental changes (temperature, pH …) and more stable sensitive elements are necessary^18^. To increase the biological entity stability, sensitive elements are usually entrapped in networks of polymeric chains, called hydrogels. Some of these, the *smart hydrogels*, show selective responsive properties to target analyte and may represent a more stable alternative to the standard biological sensing element^19,20^. Furthermore, for their ability to change their volume in response to the surrounding environment, smart hydrogel were employed in biosensors and microfluidics platforms to fabricate elements with different functions: passive elements (reservoirs, pumps, valves without power supply) have the role to drive the fluid into the reaction chamber of the sensors and active components, triggered by an external power supply, which work on demand^21^. The reversible swelling/shrinking (i.e. mass and geometrical variations) of a hydrogel is due to alteration of equilibrium electrostatic forces among the polymeric chains after concentration changes of their target in the environment^22^. In particular, pH sensitive hydrogels contain molecules with ionizable groups undergoing reversible protonation/deprotonation in accordance with variations in the environment pH^23^.

Hydrogels show a strong capability to absorb a high amount of water, and possess biological and elastic (i.e. softness) compatibility^24^: these are desirable features for biological applications in wearable chemical (bio)sensors which require mechanical flexibility to result comfortable to the body^25^. Exploiting the quartz crystal microbalance (QCM) principle, hydrogel swelling and shrinking were used to track the concentration of an analyte by mass sensing: the mass change in the smart hydrogel causes a real time shift of the QCM fundamental resonant frequency, allowing monitoring of mass with good accuracy^26,27^. Several designs of resonating MEMSs, in particular piezoelectric-based MEMSs, have been proposed and realized in combination with hydrogels^28,29^. Miniaturization has conferred several advantages with respect to the commercially available QCM, such as an improved sensitivity and lower dependence from the dimensions of the hydrogel sensing element^30^. Finally, if fabricated on polymeric substrate (e. g. Kapton, polyimide, polyethylene terephthalate, polyethylene naphthalate), resonating MEMSs also show the requested mechanical flexibility for wearable sensors^31–34^.

In this work, a wearable gravimetric sensor for sweat pH monitoring is shown. It consists of flexible piezoelectric resonators surmounted by pH responsive cylindrical hydrogel microstructures. These are made of an anionic hydrogel prepared by co-polymerization of a 10 kDa poly(ethylene glycol)-diacrylate (PEG-DA) macromer with 2-carboxyethyl acrylate (CEA), obtaining a soft and pH responsive material. The pH sensitive properties of the fabricated hydrogels are due to the carboxylic group of CEA, which is protonated in acidic conditions and deprotonated in a basic environment. The different protonation degree changes the electrostatic interactions among the polymeric chains, causing the structures to shrink or swell at different pH. Standard UV-photolithography was employed to pattern the hydrogels onto four equal piezoelectric microbalances working altogether or individually at the same resonance frequency. Each microbalance is a 1 μm-thick membrane made of aluminum nitride (AlN) sandwiched between two molybdenum (Mo) layers^35^, working as top and bottom electrodes. Kapton was used as flexible substrate and SU-8 as structural support element. Quartets of microbalances with different radii (r = 300 μm, 350 μm and 400 μm) were fabricated to obtain devices with different fundamental resonant frequencies. The characterization of hydrogel pH sensitivity was performed by optical and confocal microscopies, used to study the swelling and shrinking behavior as geometrical changes of the microstructures. Finally, characterization by laser Doppler vibrometry (LDV) in artificial sweat demonstrates the sensitivity of the fabricated flexible microbalances to acidic and basic pH conditions.

## Results

### Fabrication of flexible piezoelectric resonant (bio)MEMS

**Figure 1:**
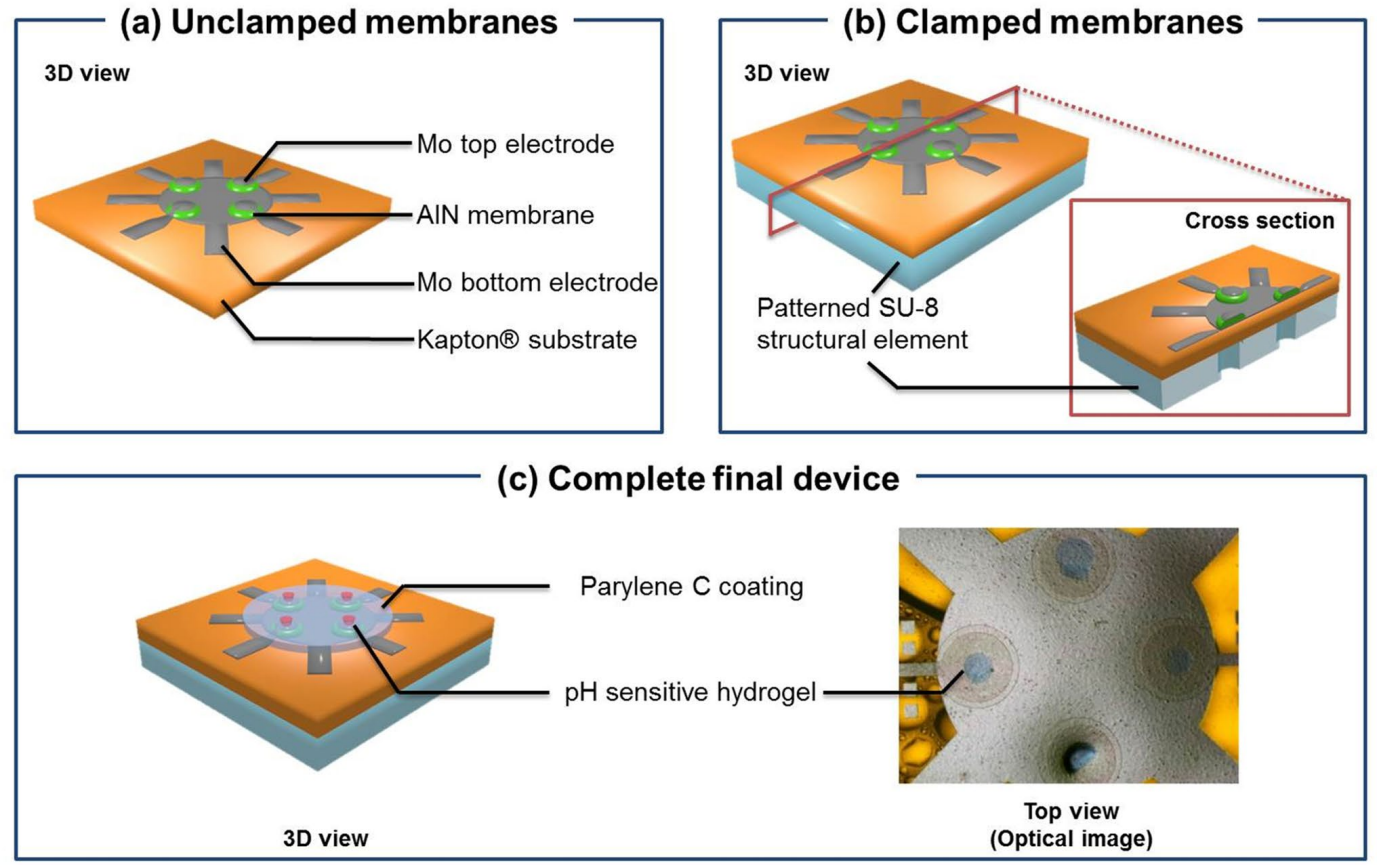
Flexible gravimetric pH sensor designs. (**a**) Computer-aided design of unclamped and (**b**) clamped membranes. (**c**) Computer-aided design (left) and optical images of complete final device.

The flexible AlN microbalances were fabricated on Kapton HN, laminated onto a rigid silicon (Si) support, starting from the protocol described in a previous work^35^. Briefly, the flexible AlN membranes were realized exploiting sputtering deposition for materials followed by UV-photolithography (Mask Aligner SUSS MA8/BA8) and etching/lift-off processes for defining electrodes and active piezoelectric elements. Each microbalance is made by a 1 μm thick AlN piezoelectric membrane (Fig. 1a, green dish) sandwiched between two 200 nm thick Mo layers (Fig. 1a, black elements), which work as top and bottom electrodes, realized on Kapton flexible substrate. The bottom electrode, shared among the four membranes, is a circular dish with a radius of 1 mm from which four metal tracks branch out. Each top electrode is a circular dish with the same radius of AlN membrane and had one metal track 45° shifted with respect the bottom metal tracks. Membranes with different radii (r = 300 μm, 350 μm and 400 μm) were fabricated in order to investigate the devices geometric characteristics. Kapton with patterned devices was detached from Si-wafer and this configuration, hereafter defined as “unclamped membrane”, is showed Fig. 1a.

The unclamped membranes were turned upside down and laminated onto a Si-support exposing its bottom side for the following SU-8 patterning step. SU-8 negative photoresist is commonly used for the fabrication of molds for soft lithography, microneedles, microfluidics and soft MEMSs devices, such as cantilevers, membranes, and microchannels^36^. Several studies have recently demonstrated its biocompatibility and reduced biofouling if compared to other common materials used for implanted medical devices^37,38^. Because of its chemical and high thermal stability, SU-8 allows the fabrication of permanent and chemically inert structures, acting as structural elements suitable in wearable (bio)sensing device. In this case, the SU-8 layer presented holes properly aligned with each membrane of the quartet: in this way each single resonator was suspended and flexible clamped membranes were obtained (clamped membranes, Fig. 1b).

The Kapton with patterned SU-8 was detached from Si-wafer, again turned upside down and laminated onto Si-support exposing its top side. Room temperature-chemical vapor deposition (RT-CVD) was used to deposit Parylene C on piezoelectric device, which was exposed to oxygen plasma and treated by 3-(trimethoxysilyl)propyl methacrylate (TMSPMA by Sigma-Aldrich) solution, obtained by a procedure described by Yuk et al^39^. Hydrogel cylindrical microstructures were obtained by UV-photolithography mask-exposition of the PEG-DA/CEA pre-polymer solution^40^. The exposed samples were placed in water:isopropanol (2:1) for 30 min to allow for complete dissolution of unreacted molecules, obtaining cylindrical hydrogel microstructures centered onto each resonator (Fig. 1c, red elements).

The final flexible piezoelectric resonator consists in a quartet of equal microbalances, which can work together or separately at the same resonance frequency, suspended on a SU-8 structural element and surmounted by pH responsive hydrogels, as shown Fig. 1c. This design allows taking the sweat from different areas from the same region of epidermis, increasing the accuracy of the pH measurements. Furthermore, it may be possible to functionalize the membranes with several kind of smart hydrogel, obtaining a multi-analytes sensor. Reasonably, an array of microbalance quartets could be implemented in order to increase the analysis area of different sweat analytes, obtaining a more reliable device.

The entire process flow is shown in Figure SI1 (Supplementary Information) which resumes and clarified each step of the flexible mass-sensor fabrication.

### Characterization of flexible piezoelectric resonant (bio)MEMS

**Figure 2:**
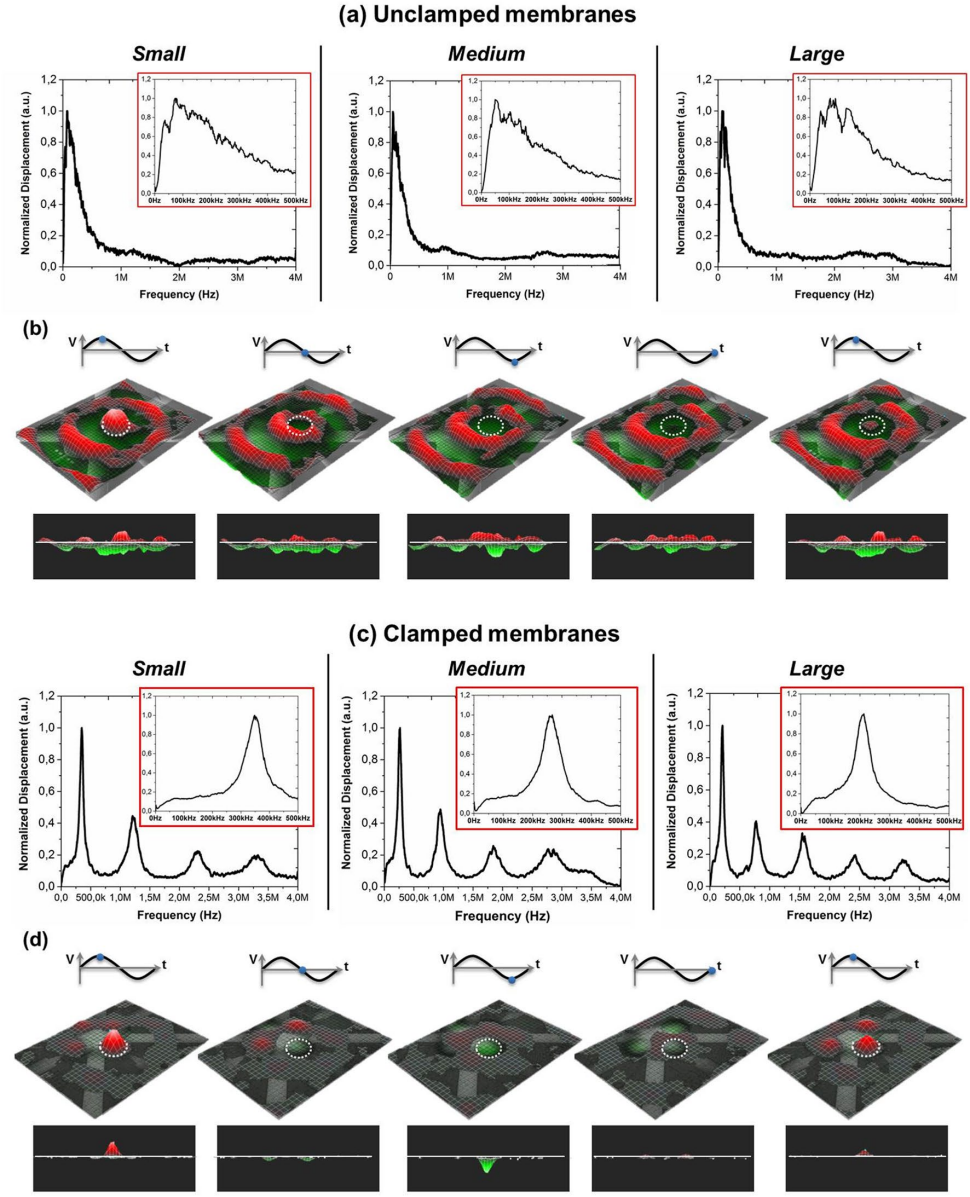
Resonant behavior studies by LDV. (**a**) Resonant frequency spectra and (**b**) LDV recorded frames of unclamped membrane at 90 kHz in one cycle of actuation: first row actuation voltage at different time, second and third row are the 3D and 2D views of the actuated membrane (highlighted by the dashed white line), respectively; (**c**) Resonant frequency spectra and (**d**) LDV recorded frames of clamped membrane at 330 kHz in one cycle of actuation: first row actuation voltage at different time, second and third row are the 3D and 2D views of the actuated membrane (highlighted by the dashed white line), respectively.

Laser Doppler vibrometry (LDV) was used to study the resonance behavior of the electrically driven membranes. Small, medium and large membranes with radii r = 300 μm, 350 μm and 400 μm, respectively, were characterized in unclamped and clamped configuration Fig. 2.

For unclamped membranes, the normalized spectra were characterized by a large frequency band below 500 kHz and no useful resonances are clearly seen for mass-sensing application (Fig. 2a). The shown behavior is due to the high flexibility of the substrate, lack of mechanical confinement, with the four membranes vibrating as a whole, leading to broad and weak resonant peaks resonances of each single membrane. In fact, as reported in Fig. 2b, after the actuation of single elements, the membrane’s vibrations can propagate through the substrate generating damping and broadening of resonant peaks.

The LDV measurements for small, medium and large clamped membranes by SU-8 structural layer are shown in Fig. 2c. In this case, it was possible to identify different resonant peaks which correspond to different vibration modes of the single membrane. The first peak corresponds to the fundamental (0, 1) mode and can be exploited for the mass-sensing measurements. Small, medium and large membranes have the first mode of vibration at 330 kHz, at 250 kHz and at 200 kHz, respectively, as expected because of it inverse proportionality with the radius of the structure^41^. The SU-8 clamping effect is shown in Fig. 2d: after the actuation of one clamped element, the vibrations cannot propagate trough the surface, remaining confined among the area of a single clamped piezoelectric membrane resonator. Therefore, the adding of SU-8 clamp caused the narrowing of the first peak and a quality factor Q ≈ 5.5.

**Figure 3:**
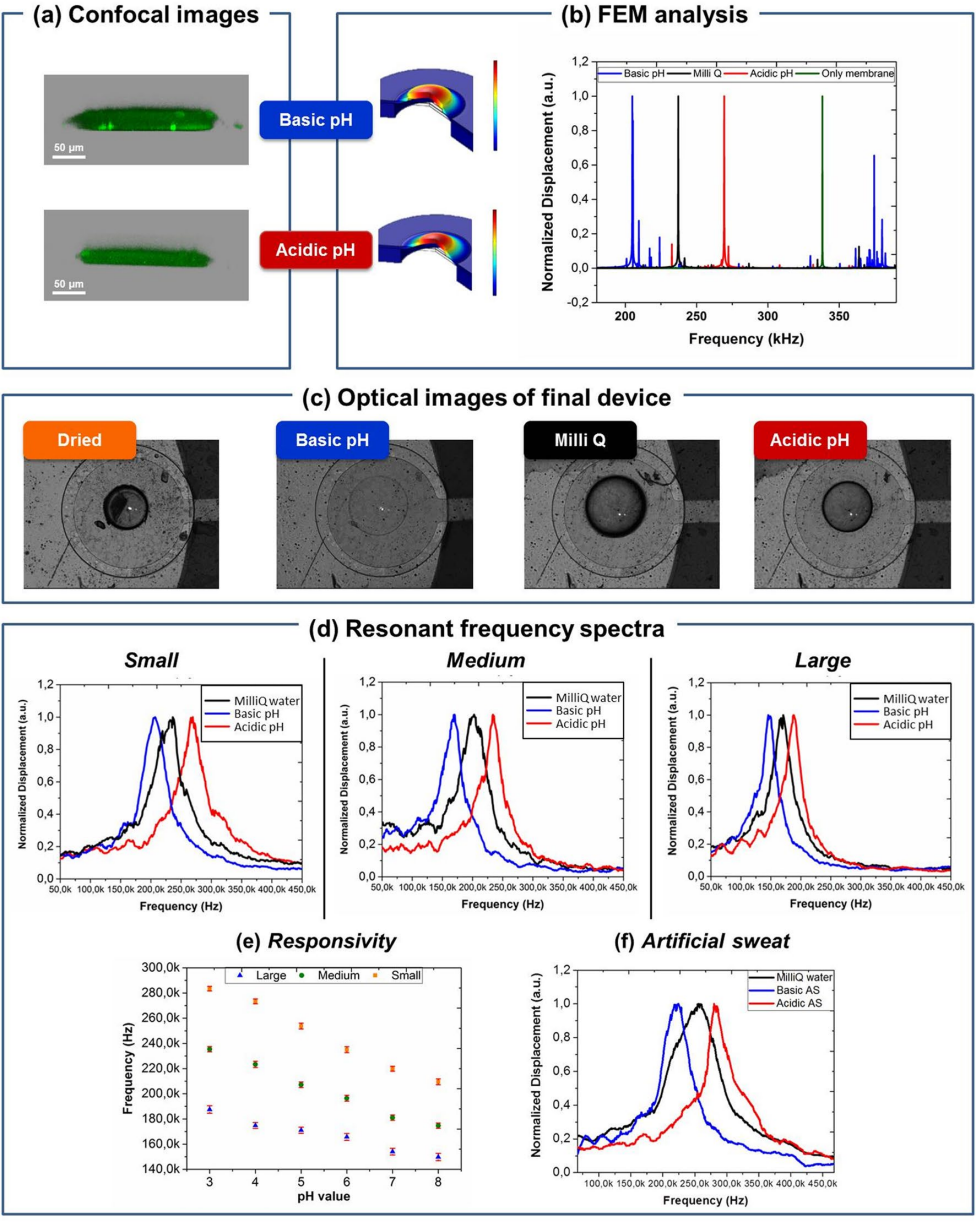
Characterization of the complete final device. (**a**) Cross-section confocal images of a swollen (top) and a shrunk (bottom) hydrogel; (**b**) FEM analysis of only membrane (green line) and of membrane surmounted by swollen hydrogel in basic buffer (blue line), Milli Q water (black line) and acidic buffer (red line); (**c**) optical images of a hydrogel in several environmental conditions (from left to right: dried with nitrogen, wetted at basic pH, Milli Q water and at acidic pH); (**d**) resonant frequency spectra of final devices; (**e**) responsivity of small (orange squares), medium (green circles) and large (blue triangles) samples and (**f**) resonant spectrum of small device using artificial sweat as buffer.

Finite element method (FEM) analyses were exploited to study the resonant behavior of the hydrogel surmounted membranes. The swelling parameters of hydrogel for FEM analysis were obtained by confocal imaging. Two cylindrical microstructures with radius 100 μm and 15 μm were firstly patterned on TMSPMA treated glass substrates (Figure SI2 and SI3, respectively). Confocal images were taken after soaking them in basic and acidic buffers and used to measure the geometric variations of swollen (basic pH) and shrunk (acidic pH) microstructures (Fig. 3a).

The measured dimensions were then employed in FEM analysis to carry out preliminary simulations about the resonant peak shift of the complete gravimetric system due to the hydrogel mass changes. The obtained resonant frequency spectra for the small (r = 300 µm) clamped membrane are shown in Fig. 3b. This picture shows the calculated deformation spectra of the clamped piezoelectric membrane at different hydrogel swelling conditions. Figure 3b (green line) shows the simulated first mode of vibration without the added mass (i.e. hydrogel): it is very close to the previously performed LDV measurements. The good agreement between the simulated and experimental data validated the FEM model for the resonant behavior investigation. Figure 3b shows also the first mode of vibration of the clamped membrane surmounted by r = 100 μm hydrogel cylinder when soaked in acidic buffer, Milli Q and basic buffer in red, black and blue lines, respectively. The lower resonant frequency was found at 205 kHz (blue line) for basic condition and the higher one at 270 kHz (red line) for acidic condition. This behavior is expected as resonant frequency of microbalances is inversely proportional to the added mass^41^. No evidence of clear variations were found in FEM studies for the smaller hydrogels (15 μm); for these reasons only the largest hydrogel cylinders were consequently patterned on piezoelectric membranes and tested.

UV-photolithography allowed to pattern hydrogels microstructures at the center of each microbalance previously coated by Parylene C. Parylene C encapsulation guaranteed electrical isolation of electrodes, protection of the skin from the contact with metals and allowed also the adhesion of pH sensitive PEG-DA/CEA microstructures on the membrane by chemical functionalization. Figure 3c shows the top view of the final device and the reversible diameter changes of the patterned hydrogel observable when it was dried under nitrogen flux, wetted with MilliQ, swollen in basic buffer and shrunk in acidic buffer. This device was characterized by LDV for defining its resonant profile. Resonant spectra were acquired under three hydrogel conditions: wetted (with MilliQ water), swollen (at high pH) and shrunk (at low pH) microstructures. In particular, for each condition, LDV measurements were conducted according to the following measurement protocol: hydrogels were firstly dehydrated by nitrogen flow directly on the final device placed on the LDV stage for few seconds and, immediately after, the measurements were acquired. A drop of the testing solution (MilliQ for wetted, basic buffer for swelled and acidic buffer for shrunk hydrogels) was directly added on the dehydrated microstructures for 10 min, the excess removed and the measurement repeated. The 10 min soaking time assured to reach chemical equilibrium in the microstructures/solution system, although a change of the dimensions of the hydrogel microstructures was visible instantaneously. LDV acquired spectra are shown in Fig. 3d in which the resonant frequency shifts for small, medium and large membranes were due to the swelling and shrinking of hydrogels microstructures, in accordance with FEM.

The measured resonant frequencies for several hydrogel conditions and for different membranes were then used to define the responsivity of each device. Responsivity is defined as the slope of the linear fit (Fig. 3e) of the resonance frequency values versus pH unit calibration curve. These data were consistent after several cycles of switch between basic to the acidic buffers, as shown by the standard deviation (red markers) at several pH units. In particular, after 40 measurements we found a standard deviation of 1.6%. 1.1% and 0.8% from the average value for large, medium and small membranes, respectively. This suggests a good stability over repeated trials, comparable to that found in literature^17,42,43^. As reported by the graphics, medium and large membranes (9 kHz/pH unit and 1 kHz/pH unit, respectively) had a lower responsivity than the one obtained by the small sample (12 kHz/pH unit). Therefore, the smaller microbalances were used to test the device in ex situ simulation of sweating. The resonant frequency changed from 220 to 280 kHz for hydrogel soaked in basic and acidic artificial sweat solutions, respectively, with a responsivity of 12 kHz/pH unit (Fig. 3f). The measured shifts suggest the potential employment of the proposed flexible gravimetric sensors for wearable monitoring of sweat pH variations.

**Figure 4:**
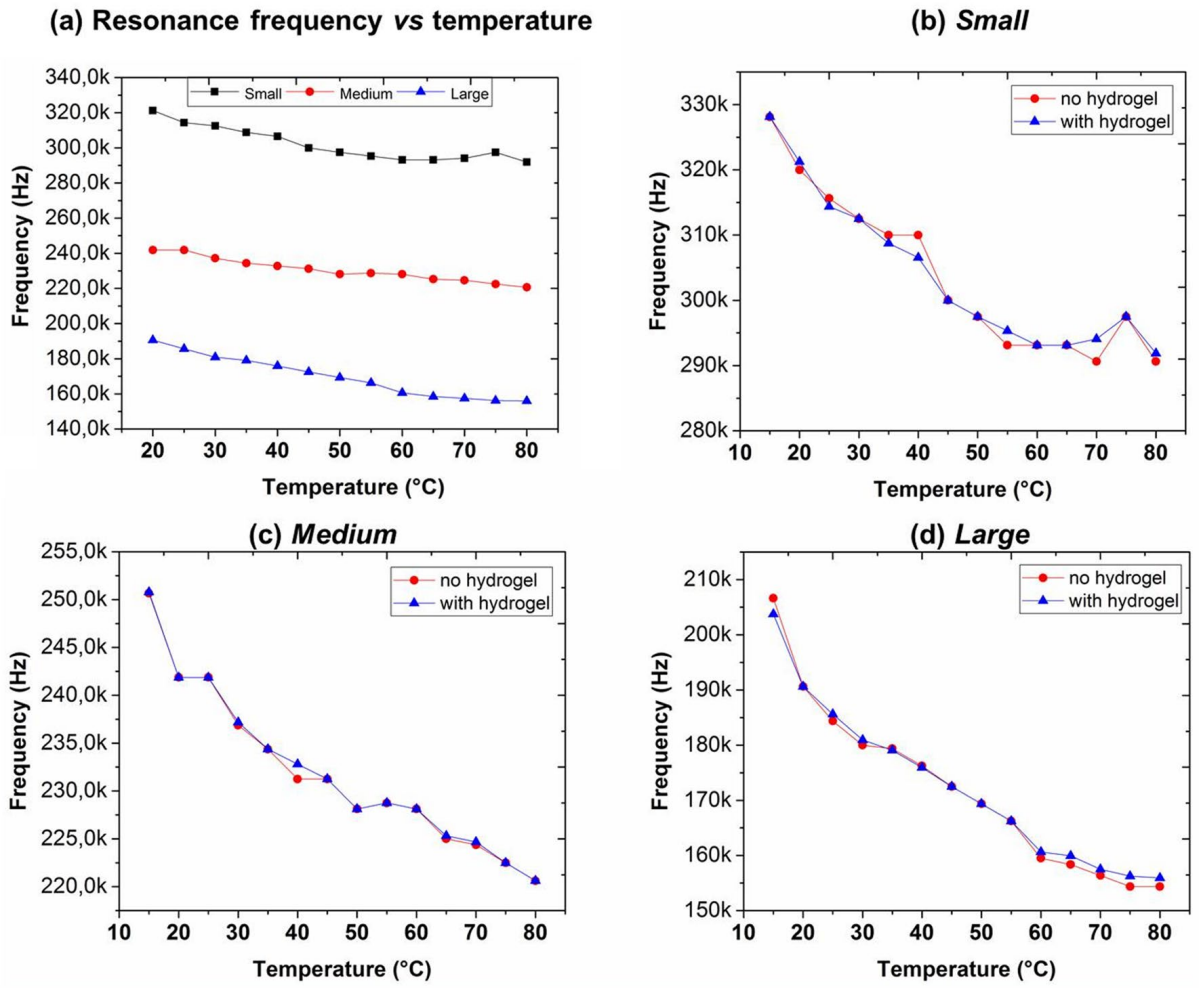
Temperature variations and resonance frequency spectra. Resonant frequency variations with temperature of small (black squares), medium (red circles) and large (blue triangles) hydrogel non-coated membranes (**a**). Comparison of (**b**) small, (**c**) medium, (**c**) large not-coated (red line/circle) and hydrogel-coated membranes (blue line/triangles).

Finally, in order to identify the relationship between the environmental temperature variations and resonance changes of the devices (Fig. 4), we added tests for calculation of temperature coefficients of frequency (TCF). In these experiments the samples were heated by a hotplate, placed under the head of laser Doppler vibrometer, and monitored by a thermal camera. The temperature was slowly (~ 1 °C/min) varied between 25 and 80 °C and frequencies spectra were acquired with a step of 5 °C by LDV measurements. TCF was calculated from the equation:

$$TCF = \frac{{\left( {f_{80} - f_{25} } \right)}}{{f_{25} \left( {80 - 25} \right)}}$$

We found a TCF of about 1301, 1597, 2907 ppm °C^−1^ for small, medium and large membranes without hydrogel functionalization, respectively (Fig. 4a). The found TCF values are comparable to those found in the literature^33^. Similar characterizations were performed for hydrogels-coated membranes without finding relevant variations with respect to uncoated TCFs (Fig. 4b, c, d). In order to correlate the temperature resonance variation with buffer temperature, we tested the microbalances dropping the same buffer at 35 °C and 40 °C, the typical extreme limits of the physiological ranges. Similar to the previous studies on environmental temperature variations, we found a resonant frequency variation correlated to the buffer temperature. In particular, we measured an average variation of about 12 kHz, 14 kHz and 13 kHz for small, medium and large microbalance in the pH responsivity from 20 to 40 °C (Figure SI4), respectively. These studies on relationship between temperature and resonance frequency variation suggest the integration of the fabricated microbalance with a temperature sensor is desirable for the future wearable application. Furthermore, the experiments on the temperature resonance variations gave stable measurements also after two months, suggesting a high stability of the device. This will allow the improvement of the device accuracy assuring reliable measurements also on skin uses.

## Discussion

In this study, a small flexible microbalance for sweat pH continuous monitoring was fabricated as new kind of wearable (bio)sensor. The system consisted of AlN-based piezoelectric membranes clamped by SU-8 structural element and equipped by responsive hydrogels for conferring pH sensitivity due to the hydrogel mass changes. Small, medium and large samples were fabricated changing the membranes radii (r = 300, 350 and 400 μm) and electrically driven for laser Doppler vibrometry (LDV) characterization in different buffer solutions. The small sample (r = 300) showed a pH responsivity of 12 kHz/pH for resonance shifting, the highest with respect to the others and was chosen for sensing characterization of the pH in artificial sweat. The membrane showed the same responsivity in basic/acidic artificial sweat solutions and proved to be suitable as a new kind of pH sensors for wearable application.

This first prototype of a flexible mass-sensitive (bio)sensor will be employed in a wearable system for continuous monitoring of pH sweat, whose variations can be linked to pathological (i.e. bacterial skin infection) or physiological status (i.e. hydration). To best of our knowledge, it is the first time a compliant gravimetric sensor for pH detection was fabricated on flexible substrate. Being characterized by small dimensions and mechanical flexibility, the proposed mass sensitive (bio)sensor can be also used for studying natural (cell, biopolymers) and synthetic (polymers) soft materials, finding a high potential application not only in new wearable and biocompatible sensing device, but also in biological and material sciences application.

## Materials and methods

### Fabrication of piezoelectric resonant (bio)MEMS

SU-8 2100 by MicroResist was deposited on cleaned bottom side of Kapton HN (by DuPont) substrate and spinned twice at 500 rpm for 30″ and 2000 rpm for 40″ to achieve a thickness of ~ 150 μm. The stacked sample was soft baked on a hotplate (65 °C for 7 min and 95 °C for 45 min) and left under chemical hood at room temperature for 45 min. The sample was exposed to UV-light in soft contact mode with a dose of 260 mJ/cm^2^, then post-baked (65 °C for 5 min and 95 °C for 12 min) and SU-8 was finally developed in a bath of SU-8 developer for 15 min.

For chemical modification of surface which allows an enduring hydrogel patterning, Parylene C coated device were treated by oxygen plasma for 10 min of 75 W powers (25 sccm, 0.6 mbar) performed by Oxygen Plasma Asher and left for 2 h in 3-(trimethoxysilyl)propyl methacrylate (TMSPMA by Sigma-Aldrich) solution, prepared mixing 100 mL deionized water, 10 μL of glacial acetic acid and 2 wt% of TMSPMA^39^. After that, the samples were removed and washed with ethanol to remove the unreacted molecules.

Hydrogel microstructures were realized by starting from the pre-polymer solution preparation, as reported in a work^40^. Briefly, 700 mg of 10 kDA PEG-DA^44^ were dissolved in 1 mL of MilliQ water and 0.5 mL of isopropanol contained Irgacure 819 (by Sigma-Aldrich) as photoiniziator (3% w_photoinitiator_/w_PEG-DA_) were addied. The mixture was stirred until the complete PEG-DA dissolution before to add CEA (30% w_CEA_/w_PEG-DA_). 700 mg of glycerol were added to the mixture for increase the viscosity of the solution and centrifuged at 14,000 RCF for 10 min in centrifugal filters (Nylon 0.22 μm) for removing air bubbles from mixtures. The pre-polymer solution was drop casted on TMSPMA treated Parylene C coating, spinned twice (I° step: 10 s, 300 rpm, 200 rpm and II° step 40 s, 1,200 rpm, 500 rpm; time, velocity and acceleration respectively) and finally exposed with a dose of 35 mJ/cm^2^ by the Mask Aligner SUSS MA8/BA8. The unpolymerized pre-polymer solution was removed putting the device in a water:isopropanol (2:1) solution for at least 30 min obtaining cylindrical hydrogel microstructures centered onto each resonator.

### Finite element method (FEM) analysis and measurements of resonance frequency

The effect of swollen/shrunk hydrogels on resonant response microbalances in air was firstly studied by finite element method (FEM) analysis and then investigated by laser Doppler vibrometer (LDV, Polytec Vibrometer MSA-500). The LDV exploits a Helium Neon (HeNe) laser source at 633 nm to generate a laser beam, directly focused on the sample surface. The unclamped, clamped and membranes surmounted by pH sensitive hydrogels were characterized by LDV, putting them on anti-vibration platform equipped with an x/y stage for moving the samples in plane directions. A chirping sinusoidal signal, in a frequency range from few Hz to 4 MHz, with voltage amplitude of 5 V was used to drive the membranes, exploiting the inverse piezoelectric effect.

### Confocal microscopy for pH responsive hydrogel microstructures characterization

In order to study the pH sensitivity of cylindrical hydrogel microstructures, confocal images were used to acquire the geometrical variations when hydrogel was soaked in different pH condition. To do that, UV-photolithographed microstructures were patterned on transparent substrate (i.e. glass) following the same procedure used for hydrogel pattering on membranes. After the patterning, hydrogels microstructures were soaked for 15 min in 10 mM phosphate buffers (pH = 8 for basic and pH = 3 for acidic conditions, respectively) and then imaged by a Leica TCS SP8 confocal microscope. Fluorescein isothiocyanate (FITC) and 4′, 6-diamidino-2-phenylindole (DAPI) were added to the acidic buffers and to Milli Q/basic buffer, respectively, to make the 3D structures fluorescent before imaging. Incident radiation wavelength at λ = 405 nm was used for structures in basic buffer for the excitation of DAPI and emission was acquired in the range 450–600 nm; radiation at λ = 488 nm was used for imaging of structures in acidic buffer and FITC fluorescence emission signal was detected between 500 and 550 nm. Changes in dimensions (thickness and radii) were measured for hydrogel microstructures with different r = 100 μm and 15 μm.

The artificial sweat was prepared by dissolving several compounds which commonly compose this biological fluid (100 µM glucose, 22 mM urea, 5.5 mM lactic acid, 3 mM NH_4_^+^, 0.4 mM Ca^2+^, 50 μM Mg^2+^ and 25 μM uric acid, 10 mM K^+^) in 10 mM phosphate buffer at the required pH for mimicking basic and acidic sweat.

## Supplementary information

Supplementary file1
