# Supplementary material for: Wearable piezoelectric mass sensor based on pH sensitive hydrogels for sweat pH monitoring
Source: Sci Rep. 2020 Jul 2;10:10854. doi: 10.1038/s41598-020-67706-y (PMC7331702; doi:10.1038/s41598-020-67706-y)
Supplement: Supplementary file 1 — Supplementary file1 [file 41598_2020_67706_MOESM1_ESM.docx]

Wearable piezoelectric mass sensor based on pH sensitive hydrogels for sweat pH monitoring

*E. Scarpa^a*^, V. M. Mastronardi^a^, F. Guido^a^, L. Algieri^a^, A. Qualtieri^a^,R. Fiammengo^a^, F. Rizzi^a*^ and M. De Vittorio^a,b^.*

^a^ Istituto Italiano di Tecnologia, Center for Biomolecular Nanotechnologies, Via Barsanti 14, 73010 Arnesano, Italy.

^b^ Università del Salento, Dipartimento di Ingegneria dell’Innovazione, via per Monteroni snc, 73100 Lecce, Italy.

*Corresponding Author

Email: [elisa.scarpa@iit.it](mailto:elisa.scarpa@iit.it) (E. Scarpa); [francesco.rizzi@iit.it](mailto:francesco.rizzi@iit.it) (F. Rizzi)

# Supporting informations

#
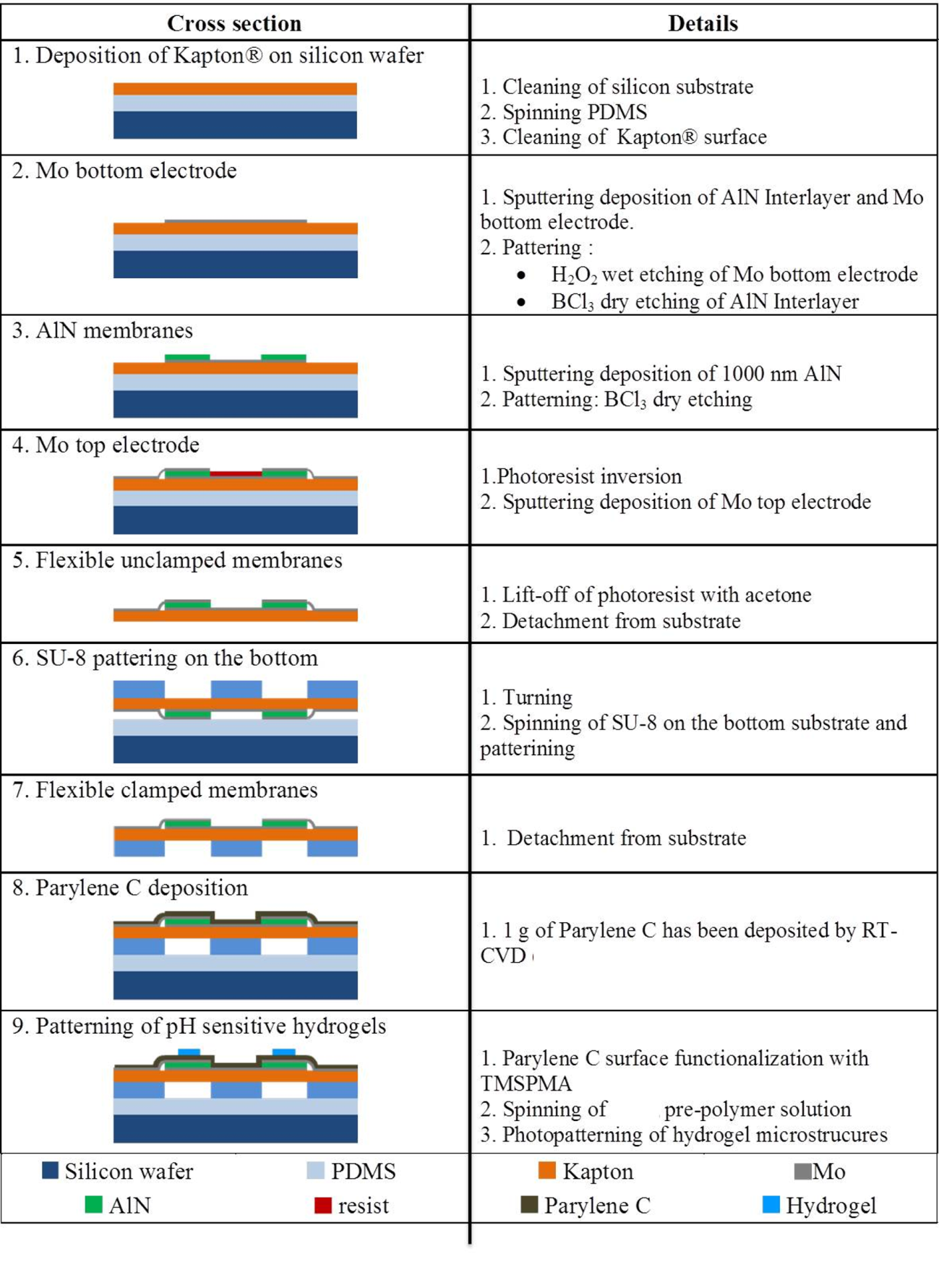


**Figure SI1: Complete fabrication protocol**


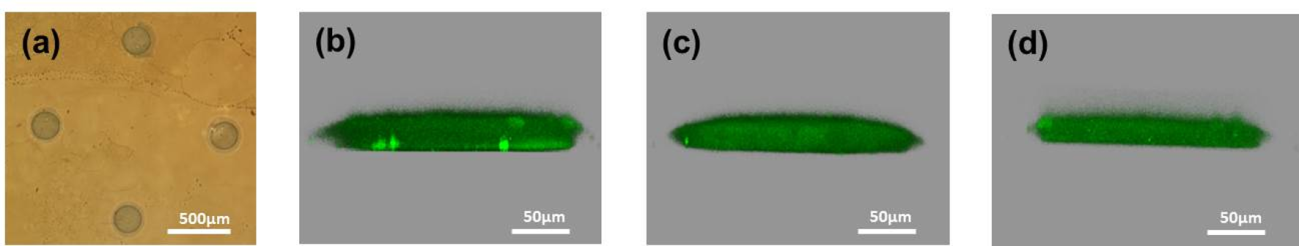


**Figure SI2: pattering of cylindrical pH sensitive structures with r=100 µm.** Optical images of the (a) big microstructures (top view). Confocal images of pH sensitive hydrogels swelled in basic buffer (b), MilliQ water (c) and acidic buffer (d) (cross-section).


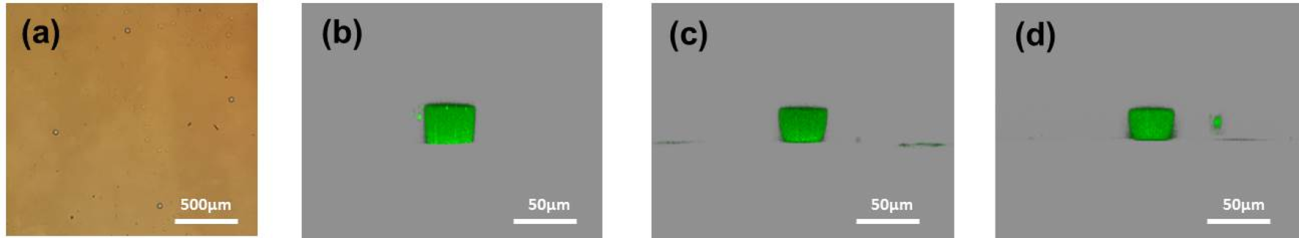


**Figure SI3: pattering of cylindrical pH sensitive structures with r=15 µm.** Optical images of the (a) small microstructures (top view). Confocal images of pH sensitive hydrogels swelled in basic buffer (b), MilliQ water (c) and acidic buffer (d) (cross-section).


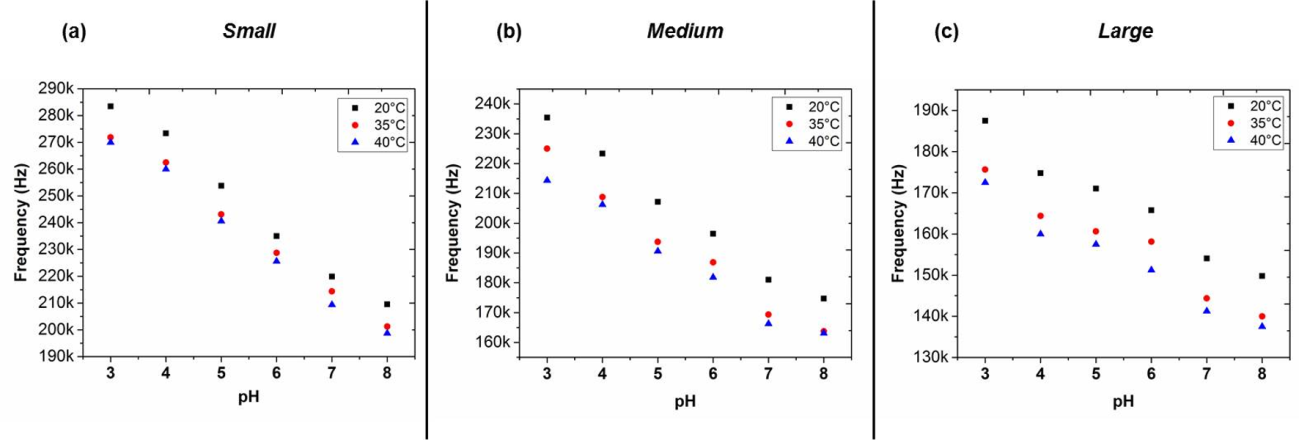


**Figure SI4: pH responsivity and variation of buffer temperature for device characterization.** Responsivity of small (a), medium (b) and large (c) samples at 20°C (black squares), 35°C (red circles) and 40°C (blue triangles) in response to pH changes.
